# Supplementary material for: Functional Analysis of the PoSERK-Interacting Protein PorbcL in the Embryogenic Callus Formation of Tree Peony (Paeonia ostii T. Hong et J. X. Zhang)
Source: Plants (Basel). 2024 Sep 26;13(19):2697. doi: 10.3390/plants13192697 (PMC11479246; doi:10.3390/plants13192697)
Supplement: Supplementary file 1 [file plants-13-02697-s001.zip › Table S1.pdf]

Table S1. Total RNA concentration and purity

Sample Name	Concentration (ng/μl)	Total (μg)	A260/A280
Seed embryo	2709.00	541.00	2.02
