# Supplementary material for: Functional Analysis of the PoSERK-Interacting Protein PorbcL in the Embryogenic Callus Formation of Tree Peony (Paeonia ostii T. Hong et J. X. Zhang)
Source: Plants (Basel). 2024 Sep 26;13(19):2697. doi: 10.3390/plants13192697 (PMC11479246; doi:10.3390/plants13192697)
Supplement: Supplementary file 1 [file plants-13-02697-s001.zip › Table S2.pdf]

Table S2. Primers and adapters

Name	Sequence
Biotin-attB2-Oligo(dT)	5′Biotin GGCGGCCGCACAACTTTGTACAAGAAAGTTGGGT(T) 3′
ATTB1-A	5′TCGTCGGGGACAACTTTGTACAAAAAAGTTGG 3′<br>3′CCCCTGTTGAAACATGTTTTTTCAACCp 5′
ATTB1-B	5′TCGTCGGGGACAACTTTGTACAAAAAAGTTGGA 3′<br>3′CCCCTGTTGAAACATGTTTTTTCAACCTp 5′
ATTB1-C	5′TCGTCGGGGACAACTTTGTACAAAAAAGTTGGAA 3′<br>3′CCCCTGTTGAAACATGTTTTTTCAACCTTp5′
pDONR222-F	5′TCCCAGTCACGACGTTGTAAAACGACGGCCAGTCTT3′
pDONR222-R	5′AGAGCTGCCAGGAAACAGCTATGACCATGTAATACGACTC3′
pBT3-STE-*PoSERK*-F	5′-AAGGCCATTACGGCCATGGTGGCGATGGAGCGAG3′
pBT3-STE-*PoSERK*-R	5′CCGGCCGAGGCGGCCGTCTAGGACCGGACAATTCGACAG3′
YFP-N-*PoSERK*-F	5′-AAGGCCATTACGGCCATGGTGGCGATGGAGCGAG3′
YFP-N-*PoSERK*-R	5′CCGGCCGAGGCGGCCGTCTAGGACCGGACAATTCGACAG3′
PoRbcL-F	5′ATGGCGTCAGCGAGTGCTAT3′
PoRbcL-R	5′TCAAACAGTAAGACCTTGTGGAGATG3′
pPR3-N-*PoRbcL*-F	5′AAGGCCATTACGGCCATGGCGTCAGCGAGTGCTAT3′
pPR3-N-*PoRbcL*-R	5′CCGGCCGAGGCGGCCAACAGTAAGACCTTGTGGAGATGGA3′
YFP-C-*PoRbcL*-F	5′AAGGCCATTACGGCCATGGCGTCAGCGAGTGCTAT3′
YFP-C-*PoRbcL*-R	5′CCGGCCGAGGCGGCCAACAGTAAGACCTTGTGGAGATGGA3′
OE-*PoRbcL*-F	5′acgggggactcttgaccatggATGGCGTCAGCGAGTGCTAT3′
OE-*PoRbcL*-R	5′aagttcttctcctttactagtTCAAACAGTAAGACCTTGTGGAGATG3′
GFP-*PoRbcL*-F	5′GGGGTACCATGGCGTCAGCGAGTGCTAT3′
GFP-*PoRbcL*-R	5′GCTCTAGAAACAGTAAGACCTTGTGGAGATGGA3′
RT-*PoRbcL*-F	5′TGGTTCACTTATCGGCTTATGTTCCTG3′
RT-*PoRbcL*-R	5′ACCACCACTTCACCTTCAATTCCTG3′
RT-*PoSERK*-F	5′GGCGAAGGAAACCACAAGAA3′
RT-*PoSERK*-R	5′CCAACGAACCATCAGCAAGG3′

RT-*PoAGL15*-F	5'GGATAATTCACTGCCGTCTT3'
RT-*PoAGL15*-R	5'CCATGCCAATTTGACTTCC3'
RT-*PoLEC1*-F	5'TCACGGCTGAGGACTTACTATGGG3'
RT-*PoLEC1*-R	5'TAATGTTCTCGGCACGCAAGGAAG3'
RT-*PoGPT1*-F	5'GCAGTTTCGTTCACCCATATCATCAAG3'
RT-*PoGPT1*-R	5'CAGCAAGAGCACATCCACCAATTATC3'
pPR3-N-F	5'GTCGAAAATTCAAGACAAGG 3'
pPR3-N-R	5'AAGCGTGACATAACTAATTAC 3'
35s-F	5'CTATCCTTCGCAAGACCCTTC3'
GFP-R	5'CCATCTAATTCAACAAGAATTGGGACAAC3'
β-TubμLin-F	5'TGAGCACCAAAGAAGTGGACGAAC3'
β-TubμLin-R	5'CACACGCCTGAACATCTCCTGAA3'
