# Supplementary material for: Functional Analysis of the PoSERK-Interacting Protein PorbcL in the Embryogenic Callus Formation of Tree Peony (Paeonia ostii T. Hong et J. X. Zhang)
Source: Plants (Basel). 2024 Sep 26;13(19):2697. doi: 10.3390/plants13192697 (PMC11479246; doi:10.3390/plants13192697)
Supplement: Supplementary file 1 [file plants-13-02697-s001.zip › Table S3.pdf]

Table S3. Positive interacting subsequence

Number	Name	Sequence
1	Protein GPR107-like	ATGTACAAGAGCACTCATATCTTCCTCTTTATTCTCCTCCTCAACTGCACCACCGCGGAGATAA<br>AAAAGCTATCATCTCCTCAGATCCTCGCCCCACAATCCTCCTCGAGAGATTCGGATTCATCATA<br>CCGGTCATGTITCCATTAAAGTCTCTTCCCCCTCTGTCACCTCCTCTAGCTCGCGGCCCAACCG<br>ACGAAGCCCTAATCCAAACCCTTCCCCCTGATCGAATCGAATGAAAACCCCCGATAGTTTTTG<br>TGTCCTCGATTCCCACTTTATTTTCCGCCTCTTCACCTTCCAAGACCTTTCCCCACCTCCTGATA<br>ACTTATTCGATGGTACCTTCAATGTCACCGATCCAAACGAGTACAGCCTCTTTTTCGCGAATTG<br>TAACCCTCAAAGCCTCGTATCAATGAAGGTTCGCGCCGAAACTTATAACCTTGATAATGGGGT<br>TAGGGACTATCTCCCTGCAGGTCAGACCCAATTGCCTTTCTGTTTGCGTTGGCTTATGTGGTTTT<br>TTTTGGGATAGGATCTTTACATGCCTCAAGAACAAATTATCTGCTCACAGGATTCATGTATTGAT<br>GGGTTTGTTGTTACTAATGAAATCTTTGAATCTATTTTGTGCTGCGGAGGATAAACATTATGTTA<br>AGGTTACAGGAACTCCTCATGGCTGGGATGTGTTGTTTTATACGTTTCAGTTCATTAGAGCGAT<br>CTTGTTGTTTACTGTGATTGTTTTGATTGGAAACGGGGTGGTCATTCTTGAAGCCATTTTTGCAA<br>GAGAAGAGAAGAAGGTGTTGATGATTGTGATCCCACTTCAGTTTTAG
2	Glucose-6-phosphatephosphate translocator (GPT1)	ATGATGTTGATCTCTTGGGCTACGAGGATTGCCGAAACCCCGAAGACAGATCTTGAATTCTGG<br>AAGACCCTTTTTCCTGTTGCTGTAGCCCATACAATTGGGCACGTAGCAGCTACTGTAAGTATGT<br>CAAAGGTTGCAGTTTCGTTCACCCATATCATCAAGAGTGGTGAGCCAGCCTTCTCCGTCTTGGT<br>TTCAAGGTTTCTCTTGGGAGAGACGTTCCCGGTGCCAGTTTATTTGTCGCTCCTTCCGATAATTG<br>GTGGATGTGCTCTTGCTGCTGTCACTGAGCTCAACTTTAACATGATTGGTTTCATGGGTGCAAT<br>GATATCAAACTTGGCGTTTTCCGCAACATATTTTCAAAGAAGGGCATGAAGGGGAAGTCTGTT<br>AGTGGAATGAACTACTACGCTTGTTTGTCCATGTTGTCTCTGTTAATTCTCACGCCGTTTGCAAT<br>TGCTGTGGAGGGACCTCAGATGTGGGCAGCTGGATTTGAAAATGCCATTTCTCAAATTGGGAC<br>CCCAGTTTCATTTGGTGGGTGGCAGCCCAGAGTATTTTCTATCATCTCTACAATCAGTGTCGTA<br>CATGTCGCTGGACGAGATCTCTCCTTACATTTAGCATTGGAATACATGAGCGATCTCTGTCATC<br>GTCTCTCTATCATCATCGTCAACACAACTGTCAGCCGGTCACGC
3	Post-GPI attachment to proteins factor 3(PGAP3)	ATGCTTGACAGAGAGAGCGAAAGAGAAGTACTTGGTAGTGGACCTGTCAAATATCATGGTAA<br>ATGGCCCTTCAGGCGTGTATATGGACTTCAGGAACCTGTTTCTGTAGCTCTATCTGCGCTCAAC<br>CTTGTAATGCACTTACATGGCTGGCTATCCTTTTTCACTTGTGCTACAAGGAGTCTCAAAAAGT<br>AGATAATGTGGCATATTTATGGGTTCTTATCAAGACAAAATATGCATACGCTAGTGAACTCATG<br>GTTTTGGAGTTCGGTTTTCCACAGTCGAGATTTATATGTGACAGAGATGCTAGACTACTCATCT<br>GCGGTGGCATTACTTGGTTTTTCACTTATACTATCTATAATAAGATGTTTCAGCGTGACCTATGA<br>GGCTGCTAGAGTCATGATTGCTGCTCCACTGGGTGCCTTTGTATCCACCCATATATTGTACCTCC<br>ACGTGTATAAACTAGATTACGGCTGGAACATGAAAGTGTGTGTTATCATGGGTGTTGCTCAACT<br>TCTCACATGGGCAATTTGGGCTGGTGTAAGTCGTCATCCTTCTCGITGGAAGGTTTGGTTGGTA<br>GTTGTCGGAGGTGGACTAGCAATGCTACTAGAGATTTATGATTTCCCTCCATATCAAGGATTTG<br>TGGATGCTCATGCTCTGGCATGCGACCACTGTCCCTCTTACCTACATTTGGTGGAGTTTCATCA<br>AAGATGATGCCATTTTCCAAACATCTGGCCTACTAAAGAAAGCAAATTAA
4	Kelch repeat-containing protein At3g27220-like	ATGCCACCGGCGCCCGTTCCTCGCCTTGATGGAGCAGCCATCCAGATCAAGAATCTTTTATATG<br>TCTTTGCAGGATATGGCACTCTTGAATATGTGCACTCTCATGTTGACATCTACAATTTCACAGAT<br>AATAAGTGGGGAGGGAGGTTTGACATGCCAAAAGAAATGGCACATTCACATTTAGGCATGGC<br>AACAGATGGGAGATACGTATATGTGGTCTCAGGACAATATGGTCCCCAATGTAGAGGTCCAGT

		GGCGCGTACATTTGTTCTGGACACCGAGACGAAAAAATGGCAGAGCATGCCTTCATTACCTGC<br>CCCTAGGTATGCTGCGGCAACCCAACTCTGGAGAGGCAGACTCCATGTGATGGGTGGTAGCA<br>AAGAGAACCGCCATACCCCCGGTTTGGAGCATTGGAGTCTGGCAGTAAAGAATGGCAAAGC<br>ACTAGAGGAAAAATGGCGGACTGAAATACCCATTCCCCGCGGAGGACCACATAGGGCTTGTA<br>TTGTAGTTGATGATCGGCTTTTTGTTATTGGTGGTCAAGAGGGTGATTTTATGGCCAAGCCTGG<br>GTCACCTATTTTCAAATGCTCGCGTAGCATGAGGTGGTTTATGGTGATGTTTATATGCTGGATAA<br>TGAGATGA
5	Transcriptional adapter ADA2-like	ATGTCTCATGTTGTTGGGAAAAATAGAAAGGAGCTTCTTGCTATGGCCAAAGGGCAGAGCGA<br>GGACAAGAAAGGGTTCTTTGCTCGGAGAACTTACATTTTTCTCCTTCAAGAGTGAAAGTTGAA<br>GAGTTATGAAGGAAGAATCTCCCATAAAAGTGGTTCTTCAGATCGGTTACCATTGAGCTTAAG<br>TGCAGAGATAGATTCTGGGGTCCGCTCGAATTGGACAAATACGAAAGGAACAGCTTCTGCCA<br>AGAAGGCATCTAACATGGCACATGTTAAGGCTGGTCCTGGTGTCATCAAAGTAGAAGATCCTA<br>AAATGGACAGGAGTTTCGGGGGGAACAAACCTAATTATTCAGGGCTTGAGGGTCCGATTGAT<br>ACGGGTGGTTATAATCCTAAAAGACAGGAGTTTGATCCTGAATATGATAATGATGCTGAGCTG<br>CTACTAGTTGACATGGAGTTCAAGGACACTGACACTGAAGATGAACGTGAGCTGAAGTTGCG<br>CGTATTACGTATCTATTATAAGAGGCTTGATGAGAGGAAGCGGAGGAAGGATTTCATACTAGA<br>AAGGAATTTGCTATATCCAAATTCTTTTGAGAGGGACTTGTCACCTGAAGAGAAGGCAATCTG<br>TCGACATTATGATGTCTTCATGCGTTTTCATTCCAAGGAAGAGCATGAGGATTTGCTTCGCACT<br>GTTATTGGAGAACACCGAACACTGAAAAGAATCCAAGAACTTAAAGAAGCTAGAGCTGCTG<br>GGTGTCGAACATCAGCCGAGGCAAATAGATACCTTGAACAGAAGAGGAAGAGGGAAACTGA<br>AGAAAGTGCCCGTAGAGCAAAGGAAAGTCCTCAGTTGGTCCAAGCAGTCTCATGGCTTCATA<br>GTCTATTGGCAGAGGATTCGAGTTGTATTACTCTAGGACAGCTACTGCCAAGTCCTGCTAATGA<br>TTTGGGTATATTGAGTTCTAGTGGGGGTACATCTACTTTTCGGAATCTGATAAACGACTATGCAT<br>GGAAATTAAGAAGACGGCCACCTTTTGTTACCGCCGGATTCTAG
6	Golgin candidate 4	ATGGCAAGGGAAGAAGCTGCTAAACTGTCAGAGTTTTTGAAGGATGCACATCAAAAAGCAG<br>AACTAACAAAGAGTGAAAAAGAAGAAATTTTAACTAAGCTTGCGCAAGCTGAGAGGCTGTT<br>AGGAGAAGGGAAAAATAGAGTAAATAAGCTCGAAGAAGATAATGGAAAACTACGG(GCCCTT<br>GAGCAGAGTATGACAAGACTTAATAGAATGTCCCTGGATTCAGATTATTTTGTTGACAGGCGC<br>ATTGTGATCAAATTGTTGGTGACGTATTTCCAGAGAAACCACAGCAAAGAGGTTTTGGATCTT<br>ATGGTTCGGATGTTGGGATTTTCTGAGGAAGAGAAGCAGAGGATTGGTGGTGCGCAGCAAGG<br>TGTAGGTGGTAAAGGTGTTGTTCGGGGAGTTTTGGGTTTTCCTGGGCGTTTTGTTGGTGGCATA<br>TTGGGAGGCAGTGGTGGTTCTGCCGAAGGACAGGCTAATGTTGCACCTGAAAATGGGTCCTTT<br>GCAGATTTGTGGGTTGATTTTCTTCTCAAAGAAACAGAAGAAAGAGAGAGGAGGGGATCTGT<br>GGAAGCTAATGCTGCTGGACCTAACCAAGAAAAAGTGCATGAAAGAAGTCCAAGTGCTGCT<br>GAACAAAGAAGCAGCGCAAGTGGTATGCCATCTGGGGTCCTCTAG
7	Probable cysteine protease RD21B	ATGTCATACCCATACGATGTTCCAGATTACGCTGGATCCACAAGTTTGTACAAAAAAGTTGGA<br>AAATTCCTCTTCTTCCTCCTCTTCATCTAGCTCTAGACATGTCGATCATCAAATACTCCGAGAAT<br>CACGGAGATAAATCAAGCTGGAGGTCCGATGAAGAAGCGATGACTTTATACGAGTCATGGCT<br>TGTAAAACACGGCAAGTCGTACAACGGGTTGGGCGAGAAGGATCGGAGGTTTCAGATCTTTA<br>AGGATAACCTTAAGTTCATCGACGACCACAACGCCGAGAATCGGAGTTATAAACTTGGGTTG<br>AACCGGTTTGCTGACCTCACCAACGATGAGTACCGGTCCATATATTTGGGGACGAAGATGGAT<br>GTTAAGAGAAGGGTGTCGAGGACAAAGAGTGATCGGTATGCTCCAAGTGTCGGCGACAGCTT<br>GCCGGATTCTGTTGATTGGAGGAAAGAAGGTGCCGTCGCTCCGGTCAAAGATCAAGGAAGTT<br>GTGGAAGTTGCTGGGCTTTCTCCACCATTGCTGCCGTGGAAGGAATAAACAAGATAGTCACTG<br>GCGACATGATCACTCTATCAGAGCAGGAATTGGTTGACTGTGATACATCCTATAACGAAGGTT

		GCAATGGAGGTCTCATGGATTATGCCTTTGAGTTCATCATCAACAATGGTGGCATTGACAGTGA<br>AGAAGATTACCCTTACCTCGGTGTTGATAGCAGATGTGACCAGTACAGGAAAAATGCCAAGG<br>TTGTTACCATTGACAGCTATGAAGATGTGCCCCAGAACGATGAGAAGGCATTACTAAAGGCA<br>GTTGCAAATCAACCCGTTAGCGTTGCCATTGAAGGTGGTGGCAGAGCATTCCAGTTATATGAA<br>TCGGGTGTGTTTACTGGAAGATGTGGAACAGCCCTAGACCACGGTGTTGCTGCTGTTGGATAT<br>GGTACTGAAAATGGTTTAATTACTGGATTGTGA
8	Endoplasmin homolog	ATGCTTCAACAACACATCACTTTGAAAACAATTAACAATAAGCTCATCCGAAAGGCTCTTGAT<br>ATGATACGTAAGCTTGCTGAACAGGATCCCGATGAGGCATATGACAAGAAAGATGTTGAGGA<br>ATCTAGCGACAATGATGAGAACAAAGGTCAATACACAAAGTTCTGGAACGAGTTTGGCAAGT<br>CTATTAAACTTGGAATCATTGAAGATGCAGCTAACAGAAACCGTTTGGCTAAACTTCTCAGAG<br>TGGAAACCACAAAGTCTGATGGTAAATTAATTTCACTAGACCAATACATCTCAAGAATGAAAT<br>CTGGACAAAAGGACATCTTTTACATAACCGGAACCACCAAGGAACAGTTGGAGAAATCACCT<br>TTTCTTTGA
9	60S ribosomal protein L4	ATGTTGAATGCTGATTTGGCTAGGATTATCAACTCCGACGAGGTCCAGTCTGTTGTGAGGCCA<br>ATCAAGAAGGAAGTGAAGAGGGCACCTCTGAAGAAGAACCCATTGAAGAATCTGAATGCGC<br>TCCTGAAGTTGAATCCATATGCCAAGACCGCAAGGAGAATGGCTCTCTTGTCAGAGGCCCAA<br>CGCGTCAAGGCAAAGAAGGAGAAGCTCGACAAGAAGAGACAACCAATCTCAAAGGAGGAG<br>AGTGCTGCCATCAAAGCTGCTGGAAAGGGGTGGTACCAGACTATGATCTCTGACAGCGATTAC<br>ACAGAGTTTGACAACTTTACCAAGTGGCTCGGAGTTTCCCAGTGA
10	FRIGIDA-like protein 4a	ATGGATTCGACTGGATTGGTAAGGTATTTGGTGGCGAAAAGGAAAGATGCAATGGCGATGAG<br>GTCAGAAATTGCTGCGGCAATGGTGGAGGGGGCGGTTGATGCGCATAGATTGGCTTTGAATGC<br>AATTGAAGAGTTTGTCGGGATGAAGGAAGCCAAGGT(CGGGTTGGCAGACAGACGGTGGGCG<br>TGTGGACTATTAGTTCAGGGGTTATTGGGGGTGAATTGGGGGTGAAGGTTGTGGCGACTAGTA<br>TCTCGGAGAGGGCGGCGAGTGTGGCAGAAAAGTGGAAGGGTATAGTGGAAGGGCAAGAAG<br>GGGACGTTGGAATGGGGGCAGCAGAAGCGGCTATGTTTCTGCAAATGGTGGTGGTATTTGGGT<br>TGAAGGAGAAGTTTGAGGAGGATTTTCTGAAGAAGTTAATAATAAGTTTTGCGACGAGGAGA<br>GACATGGCAAAGATCGCGTTGGCTATGGGGTTTGGGGAGAAAATGGGAGATATAATTGATGA<br>ATTGATGAAAAGTGGCAAGGAGATCGAGGGCTGTTTATTTTGCTTTCTGAGTCTGGTTTGA
11	Uncharacterized GPI-anchored protein At4g28100	GTGGCTCTTAGCACGATGTTAAGCAGAGACTGCCAACTAATGGGCCTGACAAACAAAACAGC<br>CTACATACGGTATCTGCTGTATTGCGCGCCATCATGTACAGTGCGCACCCTCACGAGGCCAAG<br>TGCAGTCCTGATCAAGAGAACATGCCATTAGCCGTTGATTCTCTGCAGTTTGAGAAAACACAG<br>TCATCGTCTTCTGCACCGGCTGTCGTTTTCACGTTTCCAGTTTTGCCCCTAATCGTGCTTGTAAC<br>CTTGTGTATTTTGGATTATGGCTACTTTTGA
12	Signal peptide peptidase	ATGGTGTCATACCCATACGATGTTCCAGATTACGCTGGATCCACAAGTTTGTACAAAAAAGTT<br>GGAAAGAACATCGAACGTATTGCTAATTTTGCTCTAGCAGGTTTAACATTAGCACCACTTCTTG<br>TGAAGGTAGATCCAAACTTAAATGTCATTTTGACTGCGTCCCTTACAGTTTTCGTGGGTTGCTA<br>CCGATCTGTAAAGCCAACTCCACCCTCTGAAACAATGTCCAACGAACATGCAATGCGCTTCCC<br>TTTTGTTGGGAGTGCTATGCTGTTATCATTATTCTTACTCTTCAAGTTTCTGTCGAAGGATTTGGT<br>TAATGCTGTACTGACGTGCTACTTCTTTGTGCTTGGGATCATCGCACTTTCGGCAACATTGCTTC<br>CTGCAATTAGACGTTTTTTGCCGGATCAGTGGAATCAGAATCTTATTGTCTGGCACTTTCCATAC<br>TTCCGTTCTTTGGAGATTGAGTTTACAAGATCTCAGATTGTTGCTTCAATCCCTGGAACCTTCTT<br>CTGTGCATGGTATGCTTCACAAAAGCATTGGTTGGCTAACAATATATTGGGTCTCGCATTCTGC<br>ATTCAGGGTATTGAAATGCTTTCCCTCGGATCTTTTAACACTGGTGCCATTCTCTTGGCTGGACT<br>TTTTGTTTATGACATCTTTTGGGTCTTCTTCACCCCAGTGATGGTTAGTGTTGCAAAATCTTTTG<br>ATGCTCCTATAAAGCTTCTGTTTCCGACAGCAGATATTGCACGGCCATTTTCCATGCTTGGACTT

		GGTGACATTGTAATCCCCGGCATTTTTGTTGCATTGGCCTTGAGATTTGATGTGTCGAGAGGGA<br>AACAGGGTCAATATTTTAGGAGTGCATTTCTGGGATACATGGTTGGTTTGGTCCTTACAATCGT<br>CGTTATGAATTGGTTTCAAGCTGCACAGCCTGCGCTTCTGTATATTGTACCATGTGTTATTGGAT<br>TTCTGGCTGCTCACTGCATATGGAACGGCGAAGTCAAACCGTTGTTGGAGTTTGACGAGTCGA<br>AGACTGCTAGTTCATCTGATGATGTTGGTGATGCCCAGAAGTGCATAGTCAGAAAGAGAAAG<br>GAAAATATATTCCAGCTCACTGAGAATACTGTTGTTTTTAATTGGAGGGACAAAAATGCCTTTT<br>TACTTAAACCATGGCTTAACCTA
13	Probable acyl-activating enzyme 17, peroxisomal	ATGGTAATATGGCGCAACGAAGGAGATGATGACATGCCTGTAAATAGGATGACACTTCAAGA<br>ATTGTGTTCAGAGGTTTGGTTGGTTGCTCATGCAATTAAAACACTGGGGTTGGACACAGGATC<br>TGCAATTGCAATAGATATGCCAATGCACGTCAGTTCTGTAATTATCTACCTAGCTATTGTTCTGG<br>CAGGCTATGTAGTTGTATCCATTGCTGATAGTTTCTCCAAGCGAAATATCGACAAGGCTCAAAT<br>TATCAAAAGCAAAAGCTATATTTACTCAGGAACTCATTATTCGTGGCGACAAAAGTCTACCCTT<br>GTATAGTAGAATTGTCGATGCTCAATCACCAACTGCAATTGTTATCCCAACTAGAGGCTCTAGC<br>TTCAGCGTAAAATTGCGCGAGGATGATATTTCTTGGCCTGAATTTCTTGAAAGAGTCAAAAGTT<br>TCAAAGGGGATGAGTTTGCTGCAGTAGAGCAACCTATAGAAGCCTTCACAAACATACTATTTT<br>CATCTGGAACTACAGGGGAGCCAAAAGCAATTCCGTGGACACATGCAACTCCTTTCAAGGCT<br>GCTGCAGATGGATGGTGCCACATGGATATTCGTAAAGGTGATGTTGTTGCTTGGCCCACAAAT<br>CTTGGGTGGATGATGGGTCCTTGGCTAGTTTATGCTTCATTGTTGAATGGGGCTTCCATGGCTIT<br>ATATAACGGATCACCCCTTGGTTCTGGCTTTGCTAAGTTTGTACAGGATGCTAAAGTAACTATG<br>CTTGGGGTAATCCCAAGCATTGTAAGGACATGGAAAACTACAAATTGCACAGCTGGCTACGAT<br>TGGTCTGCAATTCGTTGCTTTGGTTCGACGGGTGAGGCATCTAATGCAGATGAATACCTGTGGC<br>TAATGAGTAGAGCTTATTACAAGCCTATCATTGAGTATTGTGGTGGTACAGAAATTGTGTGA
14	Tetraspanin-8	ATGTTCCAGATTACGCTGGATCCACAAGTTTGTACAAAAAAGTTGGCTTCAATTGCCAGTCAT<br>GCAAGGCTGGATTGCTGGACAATATCAAGAGTGACTGGAAGAAGGTGGCTGTGGTCAATGTT<br>ATATTCCTCATCTTCCTCATTGTTGTCTACTCTATCGGTTGCTGTGCGTTCAGGAACAATAGGGA<br>GGATAATTCTTACCGGTTCAAGGGACACCCTTGAAGATCATCTTGAGTAG
15	Rubisco large subunit protein (rbcL)	ATGACAGGAGCTGAGTTCCAAGCCAATGATCTAGGTCTGCTCATTGAGAATACCACAGTTGAT<br>CAACTTGGAAGAAAAGTGACTATTAGCAAGGATACCACCACCATCATTGCTGATGCTGCGTCT<br>AAGGATGAAATCCAGGCTAGGATTGCACAGATTAAAAAAGAGTTGTCCGAGACGGATTCTGT<br>TTATGATTCAGAGAAACTGGCAGAGAGAATTGCCAAATTGTCGGGTGGAGTTGCTGTTATAAA<br>AGTGGGGGCTGCTACAGAAACCGAGCTTGAAGACCGTAAACTCCGTATTGAGGATGCCAAAA<br>ATGCAACATTTGCTGCCATAGAGGAAGGGATTGTCCCTGGTGGTGGTGCTGCGTTGGTTCACT<br>TATCGGCTTATGTTCCTGCAATTAAGGCTAAGCTTGAAGATCCAGATGAGAGGCTAGGTGCTG<br>ATATTGTGCAGAAGGCACTGGTATCTCCAGCATCGTTGATAGCCCAAAATGCAGGAATTGAAG<br>GTGAAGTGGTGGTGGAGAAGGTGAAGGAAAGTGAATGGGAGGTTGGTTACAATGCAATGAC<br>GGACAAGTATGAGAATTTGGTGGAATCTGGAGTTATAGATCCAGCCAAGGTGACCAGATGCG<br>CACTGCAGAATGCAGCTTCAGTTGCGGGAATGGTCCTAACTACACAGGCCATTGTCGTGGAG<br>AAGGCTAAGCCTAAAGCTCCTGTTGGTCCATCTCCACAAGTCTTACTGTTTGATTCATCACCTT<br>TTACTTAG
16	Universal stress protein A-like protein	ATGCTTGTGGAAGAAAAGAAGAAGATGAAGGTGGTGGTGGCAATTGATGAGAGCGATGGGA<br>GCTTTTATGCACTGGACTGGGTCATCAATAACATCCTGACTCGTAGGGGCGTGACACCCCATA<br>CGGCGGCGGACCTGGACTCAGCCGACATGCTTACCCTTGTCCATGTTCAGCCGCCCTTCCAGC<br>ACGCTCTTTATCCTGCCGGACCCGCCATTTATGCAGCATCTTCAGTAGTGGAGTCTGTAAGGAA<br>AGCCCAAGAACAAAATTCTGCTGCATTACTCGCTCGCGCGTTGGCTACATGCAAAGAAAAGG<br>GGGTAAAAGCAGAAACTCTTATACTTGATGGAGACCCAAAGGAAATGATATGTCAAGCTACT

		GAACAAATGCACGTTGATCTTTTGGTTATAGGCAGTCGCGGCCTCGGCACGATCAAAAGAGC<br>ATTTTTAGGGAGTGTAAGTGATTACTGTGCACATCATGCAAAATCTCCGGTCCTCATTGTGAAA<br>CCGCCAAAGTGGTGCCAAGGATCATAA
17	Tubulin beta-1 chain	ATGCGAGAAAGTGCAAAGTTCTGGGAAGTGGTTTGIGAAAGTACCAGGGCGATTCCGATCTGC<br>AGCTTGAAAGGATCAACGTGTAITACAATGAGGCAAGTGGAGTTCTTATGGATCTTGAGCCTG<br>GAACGATGGACAGCTTGAGATCTGCCTTATGGTCAGATTTTCAGGCCGGACAATTTCGTTTTTG<br>GTGTGCCGGAAACAACTGGGCAAAGGGTCATTATACAGAAGGTGCAGAGCTGATTGATTCTG<br>TGCTCGATGTTGTTCGCAAGGAGGCCGAGAACTGCGATTGCTTGCAAGGGTTTCAGGTTTGCC<br>ATTCACTAGGTGGAGGAACAGGGTCTGGCATGGGAACCCTICTTATTTCCAAGATTAGGGAGG<br>AATACCCTGATCGTATGATGCTCACTTITTCTGTATTCCCTTCTCCAAAGGTGTCTGATACAGTTG<br>TTGAGCCTTATAATGCAACTTTATCTGTTCACCAGCTTGTAGAAAATGCAGACGAGTGCATGGT<br>TCTTGATAATGAAGCTCTATATGACATTTGCTTCCGAACCCTGAAGCTCTGCAACTATGTCACC<br>ACTCCCAGCTTTGGTGATCICTGGAGTCACTTGTTGTCAGCTGAACTCTGATCTICGAAAGCTG<br>GCCGTGAACACGACTTCATTTCTTCATCTCACCTCCAGAGGTTCCCAGTCATACAGAACCGGG<br>TGGGTTTCTGTCAGTGCCAGAGCTTACCCAGCAAATGTGGGATTCAAAGAACATGATGTGTTG<br>CTGCTGACCCACGGCTGCATCTGCCAATGTTCAGGGGTAA
18	Transcription factor bHLH35	ATGTTCCAGATTACGCTGGATCCACAAGTTTGTACAAAAAAGTTGGAAAACATTACTAAGATG<br>GACAAAGCATCAATAATCAAAGACGCGATCGAGTATATCCAAGAGTTGCATCAACAAGAGAA<br>GAGAATCCAAGCTGAAATATCACAACTGGAGTCAACAAAGAAGAATTTTGATGGTTTTGATC<br>AGTCGGAGCAACAGGTTACACCATCATTGAGATCCAAGAAAAAAAGAACGGATCAGGTGCC<br>AAGTTATCAAGAATCAACAACTTCTCCCATTGAGCTGCTTGAAATACGGGTGTCTTACATGGG<br>AGAGAAGACGGTGGTAGTGAGCTTGACATGTAGTAAAAGAAGGGACACAATGGTAAAACTTT<br>GTGAGGTTTTTGAATCATTGAAGCTCAAAATTATTACTGCCAATATCACTGCTTTTTCTGGAAG<br>GCTTTTGAAAACGGTCTTTCTTGAGGCAGATGAAGAGGAGAAAGATCAGTTGAAGGCAAGG<br>ATTGAGACAGCCATAGCAGCTCTTAATGATCCACCGAGCCCTATGAGCATCTAA
19	NADPH-dependent aldo	ATGTTCCAGATTACGCTGGATCCACAAGTTTGTACAAAAAAGTTGGAGAAAGAAAAGCTGAG<br>AAATAGAGTTGAATTGGCCACTAAATTTGGTATTTGTGTGGTGGATGGAAAGACAGAGGTGCG<br>CGGCGATCCGGCCTACGTGAGGGCGTCATGCGAGGGCAGCTTGAAGAGGCTTCAAGTTAATT<br>GCATCGATCTCTATTACCAGCATCGCATCGACACCCGTGTCCCCATCGAAGTCACTTTTGGAGA<br>ACTGAAGAAATTAGTTGAGGAAAAGAAGATAAAGTATGTAGGTATATCAGAAGCTTCTGCTTC<br>AACAATCAGAAGAGCACATGCCGTTCATCCAATTACAGCTGTTCAGCTGGAGTGGTCCTTGTG<br>GTCAAGAGATGTAGAGAAAGAAATCATTCCTACTTGTAGGGAACTTGGGATTGGGATTGTTGC<br>GTACAGTCCTCTAGGACGGGGATTCTTTTCCTCAGGGCCCAAGTTTGTTGAAAACCTCACCGA<br>GGGTGATGGTAGAAAGAATTTTCCAAGGTTGAAAGGTGAGAATCTACAACATAATTTGAACTT<br>ATTTGAGCGGGTTAATGAATTGGCTGCAAGGAAGGGATGTACCCCTTCACAGTTAGCTCTAGC<br>CTGGGTTCATCACCAGGGCAACGACGTGTGCCCCATACCAGGAACCACCAAGATTCAGAACC<br>TGAACCAGAACATTGGAGCTTTGTCTGTGAAGCTAACACCAGAAGATATGGCCGAACTCGAA<br>TTCATGGCGTCTGCTAAGGGTGATAGATACCCGGTGGGGGATGTTCCTACTTGGGGAGCATTCC<br>GAAACTCCACCCTTGTCTTCATGGAAAGCGTAGACGACCCTGAGTTAGGTTATCGACTACACT<br>TCATCTATAGAAGTGATGAATAA
20	Heat shock protein 90-5, chloroplastic	ATGGAGAGGATCATGCAGTCACAAACTCTGTCTGATTCTAGCAAGCAGGCATACATGCGTGGG<br>AAGAGGGTGCTTGAGATTAACCCTAGGCATCCCATCATCAAGGAGCTTCGTGATAGAGTTGTC<br>AAGGACCCCGAGGATGAAAATGTGAAGCAAACTGCTCAATTGGTATACCAAACGGCACTGCT<br>GGAGAGCGGGTTCACCCTCACTGAACCCAAGGATTTTGCATCACGTATCTATAATTCGGTAAA<br>ATCCAGCTTAAACATCAGTCCTGATGCGGCAGTAGATGAAGAAGATGAAACAGAGGAAGTTG

	AGGCTGAGACAAAAGAAGCATCTGCTTCTACTGATGAGACAGAGACTTCTGATGTGAAGGAT GAGTTGTAG
