# Supplementary material for: Functional Analysis of the PoSERK-Interacting Protein PorbcL in the Embryogenic Callus Formation of Tree Peony (Paeonia ostii T. Hong et J. X. Zhang)
Source: Plants (Basel). 2024 Sep 26;13(19):2697. doi: 10.3390/plants13192697 (PMC11479246; doi:10.3390/plants13192697)
Supplement: Supplementary file 1 [file plants-13-02697-s001.zip › Table S4.pdf]

Table S4. Analysis of the composition and physicochemical characters of the nucleotide and deduced amino acid sequences of the PorbcL

Open reading frame (bp)	Deduced amino acid residues	Relative molecular mass (kDa)	Predicted isoelectric point (pI)	Number of acidic amino acid residues	Number of basic amino acid residues	Instability index	Aliphatic index	Grand average of hydropathicity (GRAVY)
1761	586	61.98	5.15	81	69	28.69	104.44	-0.023
